# A Non-specific *Setaria italica* Lipid Transfer Protein Gene Plays a Critical Role under Abiotic Stress

**DOI:** 10.3389/fpls.2016.01752

**Published:** 2016-11-24

**Authors:** Yanlin Pan, Jianrui Li, Licong Jiao, Cong Li, Dengyun Zhu, Jingjuan Yu

**Affiliations:** ^1^State Key Laboratory of Agrobiotechnology, College of Biological Sciences, China Agricultural UniversityBeijing, China; ^2^Life Science and Technology Center, China National Seed Group Co., LtdWuhan, China

**Keywords:** foxtail millet, lipid transfer protein, salt stress, drought tolerance, transcription factor

## Abstract

Lipid transfer proteins (LTPs) are a class of cysteine-rich soluble proteins having small molecular weights. LTPs participate in flower and seed development, cuticular wax deposition, also play important roles in pathogen and abiotic stress responses. A non-specific LTP gene (*SiLTP*) was isolated from a foxtail millet (*Setaria italica*) suppression subtractive hybridization library enriched for differentially expressed genes after abiotic stress treatments. A semi-quantitative reverse transcriptase PCR analysis showed that *SiLTP* was expressed in all foxtail millet tissues. Additionally, the *SiLTP* promoter drove GUS expression in root tips, stems, leaves, flowers, and siliques of transgenic *Arabidopsis*. Quantitative real-time PCR indicated that the *SiLTP* expression was induced by NaCl, polyethylene glycol, and abscisic acid (ABA). SiLTP was localized in the cytoplasm of tobacco leaf epidermal cells and maize protoplasts. The ectopic expression of *SiLTP* in tobacco resulted in higher levels of salt and drought tolerance than in the wild type (WT). To further assess the function of SiLTP, *SiLTP* overexpression (OE) and RNA interference (RNAi)-based transgenic foxtail millet were obtained. *SiLTP*-OE lines performed better under salt and drought stresses compared with WT plants. In contrast, the RNAi lines were much more sensitive to salt and drought compared than WT. Electrophoretic mobility shift assays and yeast one-hybrids indicated that the transcription factor ABA-responsive DRE-binding protein (SiARDP) could bind to the dehydration-responsive element of *SiLTP* promoter *in vitro* and *in vivo*, respectively. Moreover, the *SiLTP* expression levels were higher in *SiARDP*-OE plants compared than the WT. These results confirmed that *SiLTP* plays important roles in improving salt and drought stress tolerance of foxtail millet, and may partly be upregulated by SiARDP. *SiLTP* may provide an effective genetic resource for molecular breeding in crops to enhance salt and drought tolerance levels.

## Introduction

Presently, environmental problems are becoming more severe. Among the major factors, drought, salinity, and cold stresses adversely affect the growth and yield of plants (Mahajan and Tuteja, 2005). In response to abiotic stresses, plants have developed diverse signaling pathways. Various genes, including those of enzymes, transcription factors (TFs), and functional proteins that participate in mitigating the effects of stress, adjust to the cellular milieu and increase plant tolerance.

Foxtail millet (*Setaria italica*) is an important millet crop worldwide and has been cultivated in China for more than 7,000 years. Foxtail millet has a greater tolerance to drought and adverse soil conditions than maize and sorghum (Panaud, 2006). In India, China, and Japan, foxtail millet is normally cultivated in the salinity- and drought-prone regions. With remarkable drought tolerance, extensive adaptability, rich genetic diversity, and a small diploid genome about 490 Mb (Doust et al., 2009), foxtail millet provides a rich resource of stress resistance genes for study and application. Recently, considerable progress has been made on the whole-genome sequencing of foxtail millet (Bennetzen et al., 2012; Zhang et al., 2012; Jia et al., 2013), meanwhile, foxtail millet were recommended as an excellent C_4_ crop model (Doust et al., 2009; Bennetzen et al., 2012; Kumar et al., 2013; Muthamilarasan and Prasad, 2015).

Approximately 40 years ago, lipid transfer proteins (LTPs) were discovered (Kader, 1975) and defined by their ability to facilitate the transfer of phospholipids between membranes *in vitro* (Kader, 1996). LTPs are small peptides, each with eight highly conserved cysteine residues, which form the internal hydrophobic cavity of the three-dimensional structure, and an N-terminal hydrophobic signal peptide. When the N-terminal hydrophobic signal peptide is excised, the mature LTP protein targets the cell secretory pathway (Kader, 1996). Based on the molecular masses, LTPs have traditionally been classified into two families, including ∼9 kDa (LTP I) and ∼7 kDa (LTP II), respectively (Arondel and Kader, 1990; Castagnaro and García-Olmedo, 1994). Recently, based on the occurrence and distribution of non-specific LTPs (nsLTPs) in different plant species, additional subfamilies, including C, D, E, F, G, H, J, and K, were proposed (Edstam et al., 2011). The locations of the LTPs are varied. LTPs occur in the plasma membrane (Debono et al., 2009; Kim et al., 2012), cell wall (Thoma et al., 1993), or cytoplasm (Guo et al., 2013; Edstam et al., 2014). LTPs are reported to probable participated in cutin synthesis (Pyee et al., 1994; Han et al., 2001; Debono et al., 2009; Kim et al., 2012), pathogen defense responses (Maldonado et al., 2002; Silverstein et al., 2007; Guo et al., 2013; Yu et al., 2013), reproductive development (Chae et al., 2009; Zhang D. et al., 2010; Zhang Y. et al., 2010), and adaption to abiotic stresses (Guo et al., 2013; Pitzschke et al., 2014), even though their functions remain unclear.

Since the *LTP* gene induced by abscisic acid (ABA) and low temperature was discovered (Hughes et al., 1992), more LTPs responsive to abiotic stress have been found and studied. Moreover, the expression of *LTP* genes was also induced by signal molecules which involved in the signaling pathway. *Cryoprotectin* is induced by cold (Hincha, 2002), *LpLtp1* and *LpLtp2* are induced by drought (Trevino and O’Connell, 1998), *BG-14* is induced by abiotic stresses like drought, cold, and heat shock duration. It is regulated by signal molecules including ABA, anisomycin, and sphingosine, as well (Wu et al., 2004). *AZI1* from *Arabidopsis thaliana* is induced by cold and salt stresses, as well as by ethylene (Xu et al., 2011; Atkinson et al., 2013; Pitzschke et al., 2014).

In plants, the main abiotic stress responses include ABA-independent and ABA-dependent signal transduction pathways (Yamaguchi-Shinozaki and Shinozaki, 2005, 2006). Different TFs are activated in response to cold, salinity, and drought pathways, and TFs, such as DREB2A, DREB2B, bZip, MYC, and MYB, are important in responding to drought and salt stresses in several plant species (Mahajan and Tuteja, 2005; Abuqamar et al., 2009; Prasad et al., 2011; Sham et al., 2014, 2015). As functional proteins, several LTPs are regulated by upstream proteins, like TFs or kinases/phosphatases. In *Arabidopsis*, LTP3 is positively regulated by MYB96 through direct binding to the LTP3 promoter, and it is involved in plant tolerance levels to freezing and drought stress (Guo et al., 2013). AZI1, a LTP-related hybrid proline-rich protein, and a novel target of mitogen-activated protein kinase 3, is positively regulated by the latter, and plays a role in salt tolerance in plants (Pitzschke et al., 2014).

In this study, we isolated a novel nsLTP gene (*SiLTP*) from a *S. italica* suppression subtractive hybridization (SSH) cDNA library, and investigated its biological functions. The transcription levels of *SiLTP* were induced by NaCl, polyethylene glycol (PEG) and ABA. The expression of *SiLTP* enhanced the salt and drought tolerance levels of transgenic tobacco and foxtail millet. SiARDP which plays important role in the abiotic stress response (Li et al., 2014) could bind to the DRE element of *SiLTP* promoter region. Moreover, *SiLTP* transcription level was upregulated in *SiARDP*-OE (overexpression) foxtail millet. *SiLTP* plays important roles in response to salt and drought stresses in foxtail millet, and may be a candidate of SiARDP downstream genes.

## Materials and Methods

### Plant Materials and Treatments

Foxtail millet (*S. italica* cv. Jigu11) was cultivated in the greenhouse or growth chamber. The condition is that the temperature is 25°C and the photoperiod is 16 h/8 h (light/dark). For SSH library construction, 21-day-old seedlings were pre-cultured for 3 days in 1/3 Hoagland liquid medium with bubbling. Then, seedlings were transferred to the same medium applied with 20% (m/v) PEG 6000 (Sigma-Aldrich, Shanghai, China) or 250 mM NaCl, respectively. Meanwhile, plants cultured in the medium without any addition as control. After treatment with PEG or NaCl for 0, 1, 3, 6, 12, and 24 h, the roots and shoots were separately stored at -80°C for RNA extraction. To analyze the *LTP* expression pattern, foxtail millet roots, stems, leaves, inflorescences, and seeds of different developmental stage: 5, 10, and 15 days after pollination, were collected and stored at -80°C until use. To confirm *SiLTP* expression levels under stress, 14-day-old foxtail millet seedlings were subjected to 1/4 Hoagland liquid medium containing 100 mM NaCl, 20% (m/v) PEG 6000 or 10 μM ABA, independently, for the indicated time. Harvested seedlings were immediately frozen and stored at -80°C until use.

*Arabidopsis thaliana* (Col-0) were grown in the growth chamber at 21–22°C under 16 h/8 h (light/dark) conditions for transformation or GUS staining.

### Suppression Subtractive Hybridization

Total RNA was extracted from foxtail millet seedlings using a hot phenol method (Longeman et al., 1987). mRNA was purified from total RNA using a PolyATtract mRNA Isolation System (Promega, Madison, WI, USA). Then, 4 μg mRNA mixed with equal amounts of roots and shoots mRNA of the seedlings at indicated times after independent NaCl and PEG treatments, as well as from non-treated seedlings. These samples were the testers and drivers in the construction of the SSH cDNA library. A Clontech PCR-Select cDNA Subtraction Kit (Clontech, Palo Alto, CA, USA) was used to construct the library. After first round PCR, the secondary round PCR was carried out and the products were ligated to the vector of pGEM T-easy (Promega). The ligation product was transferred into the competent cells of *Escherichia coli* DH5α. Colonies growing on the medium were collected and cultured at 37°C for 12 h. Then, plasmids were extracted from the *E. coli* and digested by *Not*I. The resulting fragments between 800 and 2,000 bp were recovered, and inserted into the plasmid 19-T (Promega). The resultant cDNA libraries were transformed to *E. coli* DH5 and sequenced.

### RNA Extraction, Semi-qRT-PCR, and qPCR

Total RNA extracted by TRIzol reagent (Invitrogen, USA) was digested with RNase-free DNaseI (Takara, Japan), and revised transcribed into first-strand cDNA using MLV-Reverse Transcriptase (Promega). For quantitative real-time PCR (qPCR), a 20-μL reaction system, which containing 10 μL 2 × SYBR mix (CoWin Biotech, Beijing, China) and 100 ng of cDNA template, was adopted. Primers of the *SiLTP* gene (GenBank: LOC101782694) and the reference *Actin* gene (GenBank: AF288226) are shown in Supplementary Table S1. qPCR was conducted by the Lightcycler 480 Real-Time PCR System (Roche, Indianapolis, IN, USA). Thermal Cycler as we described before (Pan et al., 2015). Each qPCR was run in three biological replicates. The ΔΔCT method was adopted for the calculation of relevant genes expression levels (Bustin et al., 2009). Data represent the means and standard deviations (SD) of three replicates. To analyze *LTP* expression patterns, semi-quantitative reverse transcriptase PCR (semi-qRT-PCR) was conducted, with the following cycling parameters: 94°C for 1 min, followed by 25 cycles of 94°C for 1 min, 60°C for 30 s and 72°C for 30 s (Pan et al., 2015). Total RNA was extracted from 30 plants at the indicated times after the treatments.

### Subcellular Localization of SiLTP

*SiLTP* coding region was amplified with the primers (Supplementary Table S1) without the terminating codon. The PCR products were digested with *Xba*I/*Kpn*I and inserted into the corresponding sites of *pSuper1300-GFP.* The constructed *pSuper1300-LTP-GFP* was used for *Agrobacterium*-mediated transient transformation of tobacco epidermal cells (Zhang Y. et al., 2010). *pSuper1300-GFP* was used as the control. Maize protoplasts were prepared and transformed as described by Wang et al. (2014). Green fluorescent protein (GFP) signals were observed using a confocal laser scanning microscopy (LSM 510, Carl Zeiss MicroImaging GmbH, Jena, Germany).

### Generation of Transgenic Plants

The coding region of *SiLTP* was amplified and ligated into the *Xba*I/*Kpn*I sites of *pCAMBIA2300* and the *Sal*I/*Kpn*I sites of *pCOU* to generate the constructs *pCAMBIA2300-SiLTP* and *pCOU-SiLTP*, respectively. The forward and reverse sequences of *SiLTP* were connected with the 400 bp GUS DNA fragment spacer, and then inserted into the *Bam*HI/*Sac*I sites of *pCOU* to generate the *pCOU-SiLTP-RNAi* (RNA interference) vector. *pCAMBIA2300-SiLTP* was introduced into the *Agrobacterium tumefaciens* strain LBA4404 and transformed into tobacco. The constructs *pCOU-SiLTP* and *pCOU-SiLTP-RNAi* were transformed into foxtail millet mediated by *A. tumefaciens* strain LBA4404 as described previously (Qin et al., 2008; Wang et al., 2011; Pan et al., 2015). The transgenic tobacco and foxtail millet plants were confirmed by PCR. The expression of *SiLTP* in transgenic plants was determined by semi-qRT-PCR and qRT-PCR using *SiLTP*-specific primers (Supplementary Table S1). *Actin* was used as the endogenous reference. In order to analyze the *SiLTP* promoter, we amplified the putative *SiLTP* promoter with the primers (Supplementary Table S1) and inserted into the *pCAMBIA1391-GUS*. The construct *pCAMBIA1391-proLTP::GUS* was then introduced into *A. tumefaciens* strain GV3101, and then transformed into *Arabidopsis* (Clough and Bent, 1998). Seeds were obtained following self-pollination.

### Seed Germination Assay of SiLTP Transgenic Tobacco and Foxtail Millet under Abiotic Stress

Seeds of wild type (WT) and T_1_ transgenic tobacco plants were sterilized by chlorine gas (Clough and Bent, 1998) and spread on 1/2 Murashige and Skoog (MS) medium supplemented with 0, 200, or 250 mM mannitol, or 100 mM NaCl, independently, and were grown for 10 days in a growth chamber under the conditions of 25°C and 16 h/8 h (light/dark) photoperiod. The percentage of seeds germinated from each sample by the 10th day was calculated, and the lengths of seedling roots and shoots were measured.

Both the WT and T_2_ transgenic foxtail millet seeds were germinated on filter papers moistened with water containing 0, 100, 150, or 250 mM NaCl, or -0.5 MPa PEG at 25°C in the darkness, independently, and the following formulae were used to calculate the germination stress index (GSI). The promptness index (PI) = nd2 + 3/4 (nd4) + 1/2 (nd6) + 1/4(nd8), the nd2, 4, 6, and 8 indicate the percentage of seeds observed to germinate after 2, 4, 6, and 8 days, separately, and the GSI = (PI of stressed seeds/PI of control seeds) × 100 (Bouslama and Schapaugh, 1984). The shoot and root lengths of seedlings were measured on the 8th day after sowing.

### Abiotic Stress Tolerance of SiLTP Transgenic Tobacco and Foxtail Millet

Four-week-old seedlings of WT and T_1_ transgenic tobacco were irrigated with water supplemented with 250 mM NaCl every 4 days, or not irrigated for 21 days, and then re-watered and grown for 3 days, the phenotypes of transgenic lines and WT were investigated and surviving plants were counted. Two-week-old foxtail millet seedlings were irrigated with water supplemented with 100 mM NaCl every 4 days, or not irrigated for 10 days, and then re-watered and grown for 3 days. The phenotypes were investigated and surviving plants were counted. The above-ground parts of seedlings at the indicated times of stress treatments were collected and used in subsequent proline and sugar measurements.

For the heading stage drought treatment, foxtail millet was grown in the greenhouse. After the 8th or 9th leaf appeared, the plants were not irrigated for 28 days, then re-watered and grown for 3 days, the survival rates were calculated.

### Proline Content Measurement

The free proline contents were measured as described by Bates et al. (1973) and Wang et al. (2014). 0.05 g dry leaf tissue was used. Free proline was extracted with 3% sulphosalicylic acid at 95°C for 15 min. Two milliliters of supernatant was transferred to a new tube, 2 mL of acetic acid and 2 mL of acidified ninhydrin reagent were added and mixed well. The reaction was carried out at 95°C for 30 min. After that, 5 mL of toluene was added under continuously shaking. The toluene layer were fetched and measured at 520 nm for absorbance.

### Soluble Sugar Content Measurement

Soluble sugar contents were examined according to Yemm and Willis (1954) and Wang et al. (2014). 0.1 g dry leaf tissue was used for the extraction of soluble sugar. Ten milliliters of 80% ethanol was added and the reaction was conducted at 80°C for 30 min with constant stirring. After filtration, 1 mL extracting solution was transferred to a new tube, and incubated in a boiling water bath to evaporate ethanol. Next, water was added to make the final volume of this solution up to 1 mL. Then 5 mL 0.15% anthrone solution were added, mixed well, and incubated for 15 min at 95°C. The reaction solution were cooled to room temperature and measured the absorbance at 620 nm. Glucose was used for making standard curve.

### Statistical Analysis

For stress tolerance analysis, the experiment was repeated three times, and each time with three replicates. For Proline and sugar content measure, each data point had three replicates. Data represent the means and SD of three replicates, respectively. Student’s *t*-test were analyzed by the GraphPad Prism 5 software.

### Histochemical GUS Staining

In transgenic *Arabidopsis*, GUS expression were detected by histochemical GUS staining (Jefferson et al., 1987; Pan et al., 2015). Chlorophyll was removed from tissues in 70% ethanol after the GUS staining, then the tissues were photographed in a dissecting microscope (Olympus SEX16, Tokyo, Japan).

### Electrophoretic Mobility Shift Assay

*SiARDP-GST* and *SiAREB1-GST* vectors were constructed and transferred into *E. coli* (BL21) by Li et al. (2014). After induction, the fusion proteins were purified trough Glutathione Sepharose 4B column (GE, USA). Oligonucleotides (P1-R, P2-R, and P3-R) and their reverse complementary oligonucleotides (P1-F, P2-F, and P3-F, respectively), which were labeled with biotin, were synthesized. Meanwhile, the oligonucleotides (competitor2-1R, competitor2-1F, competitor2-2R, and competitor2-2F) for competitors were synthesized. All of these sequences are shown in Supplementary Table S1. Double-stranded DNA was obtained by heating oligonucleotides at 92°C for 30 s and annealing at 30°C overnight. The gel-shift assay was performed following the manufacturer’s protocol for the LightShift Chemiluminescent Electrophoretic Mobility Shift Assay (EMSA) Kit (Thermo, USA).

### Yeast One-Hybrid Assay

The bait oligonucleotides of DRE2 (DRE2-F and DRE2-R) and the mutant bait oligonucleotides (mDRE2-F and mDRE2-R) were synthesized (Supplementary Table S1). All of the bait and mutant bait sequences were inserted into the pAbAi vector at the *Hin*dIII and *Xho*I sites to create the bait vectors. The bait vectors were transformed into yeast strain Y1HGold following the protocol of the Yeastmaker Yeast Transformation System 2 (Clontech, USA), and the transformed yeast was cultivated on synthetic medium without uracil but supplied with various contents of aureobasidin A. Then, *SiARDP* prey vector constructed by Li et al. (2014) was transformed into bait strains. The medium without leucine and with 500 ng mL^-1^ aureobasidin A was used. The transformed bait strain was cultivated at 30°C for 3 days.

## Results

### Characterization of *SiLTP* and its Expression Pattern in Foxtail Millet

SSH libraries was constructed using foxtail millet “Jigu 11” seedlings independently treated with PEG and NaCl. The *SiLTP* was cloned from this library. It contains 282 nucleotides, and the deduced protein contained 93 amino acid residues. The tertiary structure of SiLTP was predicted using SWISSMODEL (Arnold et al., 2006), and nsLTP2 from rice (*Oryza sativa*) is the most appropriate structural template for SiLTP. Between nsLTP2 and SiLTP, 64.7% of positional sequence identity and 53% similarity reflected the high degree of matching between the nsLTP2 model and SiLTP. SignalP 3.0 (Bendtsen et al., 2004) indicated that there is a signal peptide containing 25 amino acid residues in SiLTP which is located at N-terminal and hydrophobic. After cleavage of the signal peptide, a mature protein of 68 amino acids was produced. The predicted molecular weight is 6.9 kDa. This indicated that *SiLTP* belongs to the LTP II subfamily.

**FIGURE 1:**
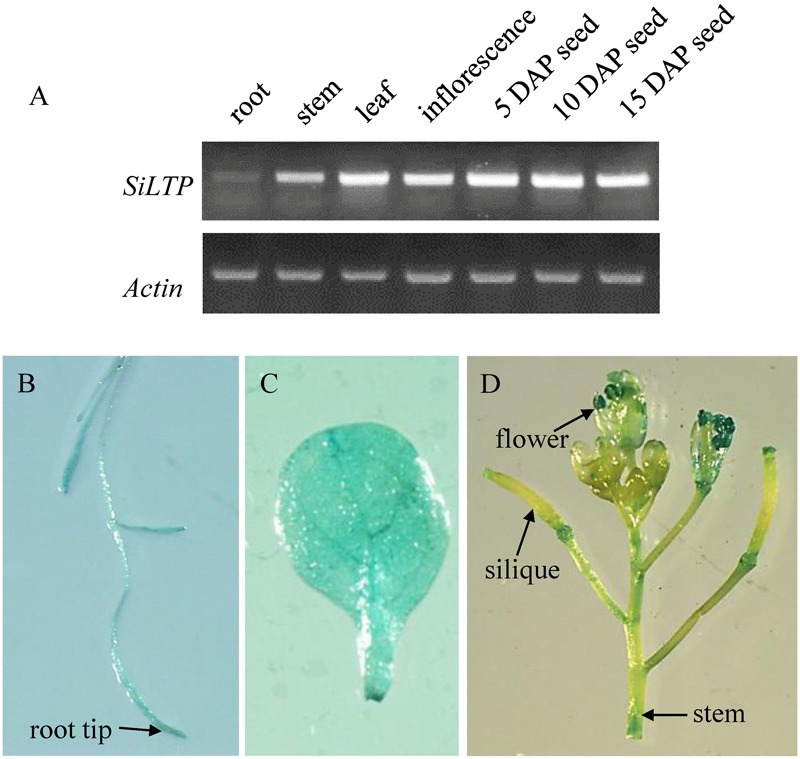
**Expression pattern of *SiLTP*. (A)** Semi-qRT-PCR analysis of *SiLTP* in various foxtail millet organs. **(B–D)** Histochemical analysis of the SiLTP promoter::GUS expression in a transgenic *Arabidopsis* root **(B)**, 10-day-old leaf **(C)**, and inflorescence **(D)**.

**FIGURE 2:**
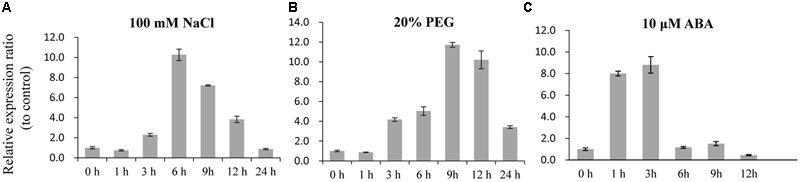
***SiLTP* transcript accumulation in response to various treatments.** Two-week-old foxtail millet seedlings were treated independently with 100 mM NaCl **(A)**, 20% (m/v) PEG 6000 **(B)**, and 10 μM ABA **(C)** for the indicated times.

To analyze the *SiLTP* expression pattern, total RNAs were extracted from seeds of different developmental stage, roots, stems, leaves, inflorescences which collected before, and used for semi-qRT-PCR. *SiLTP* was detected in all of the tested tissues. However, the transcription level of *SiLTP* was lower in roots compared with other tissues (**Figure 1A**). Additionally, the *SiLTP* promoter was cloned and fused with GUS. The construct *p1391-proSiLTP::GUS* was transformed into *Arabidopsis*, 13 transgenic lines were obtained, and three T2 transgenic lines were selected for GUS staining. The GUS signals were detected in the root tips, stems, leaves, anthers, and both ends of siliques (**Figures 1B–D**). qPCR was carried out to determine the expression levels of *SiLTP* under stress conditions. The maximum *SiLTP* transcript level was detected 6 h after the salt treatment began, and then gradually decreased (**Figure 2A**). The PEG treatment resulted in the maximum *SiLTP* accumulation level, which was 12-fold 9 h after the treatment began (**Figure 2B**). The *SiLTP* transcript level increased significantly 1 h after the ABA treatment began, reached its highest level (ninefold) after 3 h, and then decreased (**Figure 2C**). These results imply that *SiLTP* is involved in plant responses to abiotic stresses through an ABA-dependent pathway.

### Subcellular Localization of SiLTP

**FIGURE 3:**
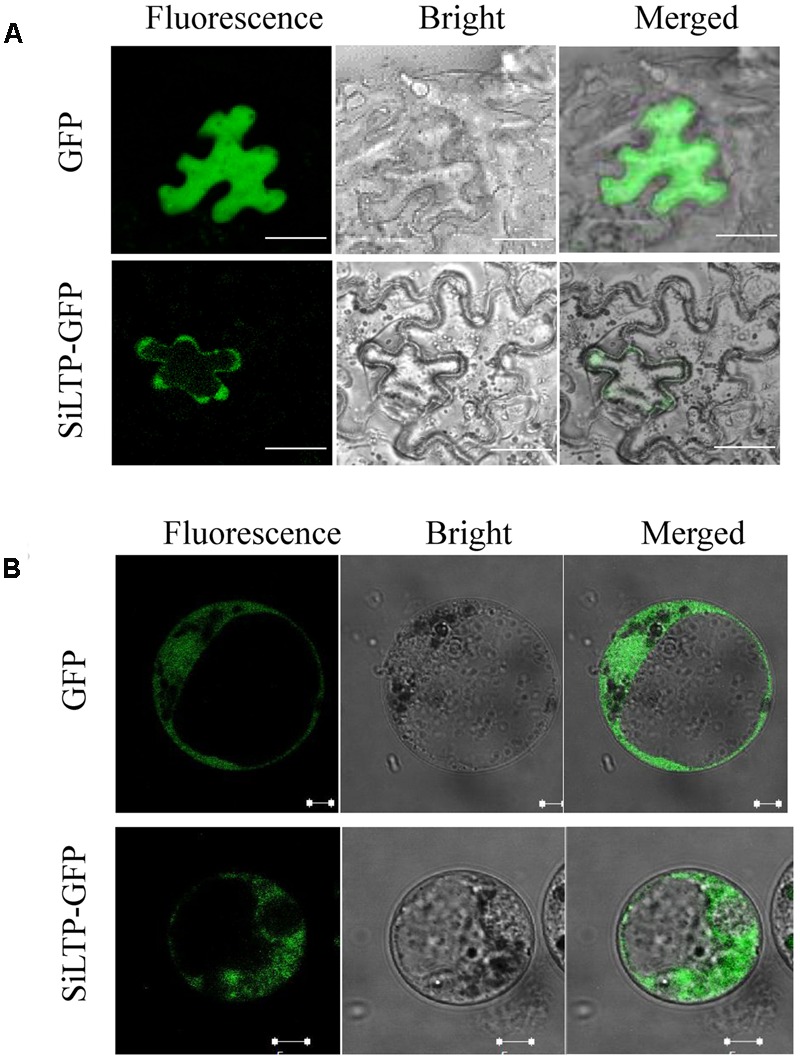
**Subcellular localization of SiLTP in tobacco epidermal cells (A)** and maize protoplasts **(B)**. The green fluorescence signals, bright-field, and an overlay of the green fluorescence and bright-field images are shown from left to right. **(A)** Bar = 50 μm; **(B)** bar = 5 μm.

To determine the subcellular localization of SiLTP, the *pSuper::SiLTP-GFP* construct was generated and transformed into *Nicotiana benthamiana* epidermal cells. The GFP fluorescence of *pSuper::SiLTP-GFP* was distributed in the cytoplasm, especially the cytoplasm near the cell membrane, and the fluorescence of control cells containing *pSuper::GFP* was ubiquitously localized (**Figure 3A**). To further confirm these results, maize protoplasts were subsequently used. The GFP fluorescence was observed throughout the cytoplasm (**Figure 3B**). Thus, SiLTP was localized in the cytoplasm.

### Ectopic Expression of *SiLTP* Improves the Abiotic Stress Tolerance of Transgenic Tobacco

**FIGURE 4:**
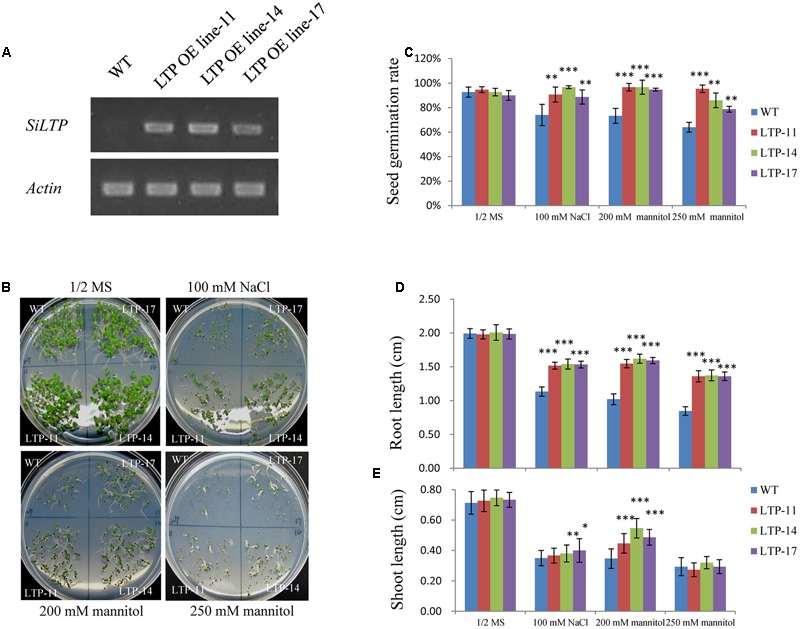
**Effects of NaCl and mannitol stresses on *SiLTP* transgenic tobacco seed germination.** Expression levels of *SiLTP* in transgenic tobacco and WT plants **(A)**. NaCl or mannitol sensitivity of WT and transgenic plants **(B)**. Seeds of WT and transgenic lines were germinated and then grown for 8 days on medium independently supplemented with 0 (control), 100 mM NaCl, 250 mM mannitol, and 300 mM mannitol. The corresponding seed germination rates **(C)**, root lengths **(D)**, and shoot lengths **(E)** are compared. ^∗^, ^∗∗^, and ^∗∗∗^ indicate statistically significant differences at *P* < 0.05, *P* < 0.01, and *P* < 0.001 (Student’s *t*-test), respectively.

**FIGURE 5:**
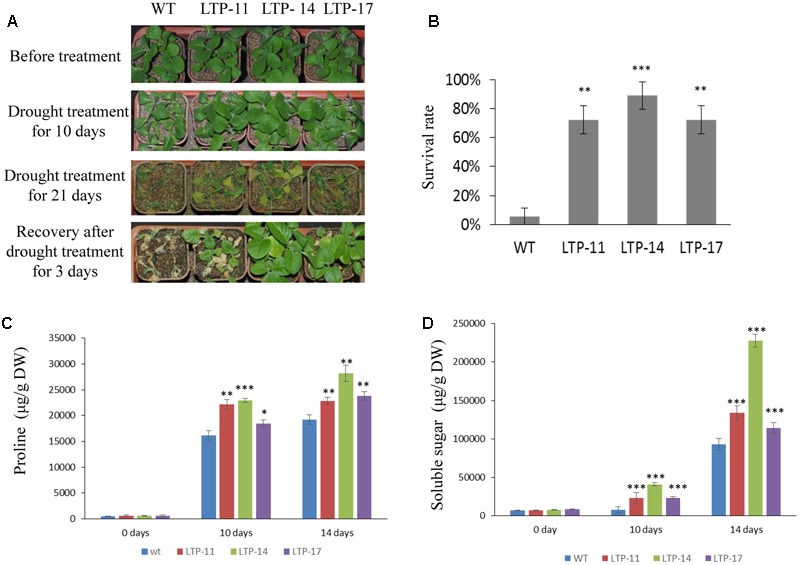
**Drought resistance analysis of T1 transgenic and WT tobacco seedlings.** Four-week-old seedlings of transgenic lines and WT were not watered for 0, 10, and 21 days, and were then re-watered **(A)**; the survival rates of transgenic and WT seedlings after re-watering **(B)**; the proline content **(C)**, and soluble sugar content **(D)** were analyzed in transgenic and WT plants after exposure to drought for 10 and 14 days, respectively. Six seedlings of each transgenic line and WT were planted in each small basin for the drought treatment and corresponding analysis; 18 plants were used in each experiment. ^∗^, ^∗∗^, and ^∗∗∗^ indicate statistically significant differences at *P* < 0.05, *P* < 0.01, and *P* < 0.001 (Student’s *t*-test), respectively.

**FIGURE 6:**
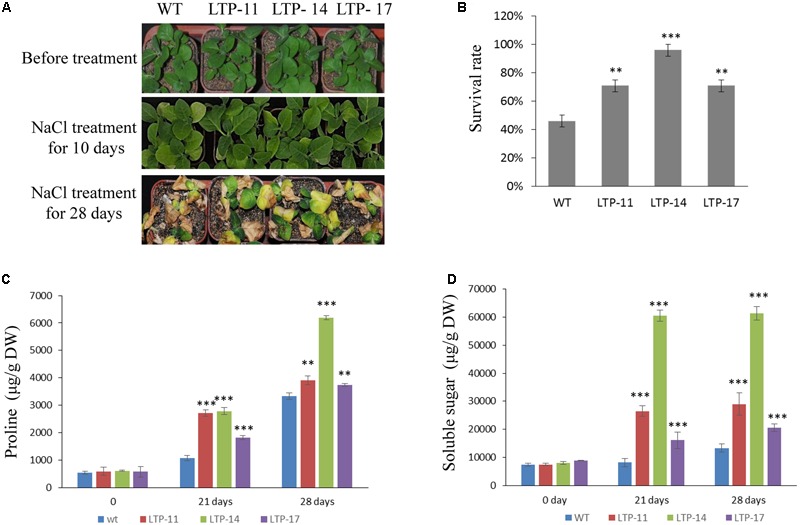
**Salt resistance analysis of T1 transgenic and WT tobacco seedlings.** Four-week-old seedlings of the transgenic lines and WT were irrigated for 0, 10, and 28 days with 250 mM NaCl **(A)**; the survival rates of transgenic and WT seedlings after watering for 28 days with NaCl **(B)**; the proline content **(C)**; and soluble sugar content **(D)** were analyzed in transgenic and WT plants after watering for 21 and 28 days with NaCl, respectively. Six seedlings of each transgenic line and WT were planted in each small basin for the NaCl treatment and corresponding analysis; 18 plants were used in each experiment. ^∗∗^ and ^∗∗∗^ indicate statistically significant differences at *P* < 0.01 and *P* < 0.001 (Student’s *t*-test), respectively.

Transgenic tobacco plants were generated using the construct pCOMBIA2300-SiLTP, and three independent T1 transgenic lines (LTP-11, LTP-14, and LTP-17) with high expression levels were chosen for further analysis (**Figure 4A**). To investigate the influence of salt and drought stress on seed germination and growth, the WT and transgenic tobacco seeds were spread on 1/2 MS medium supplied with various contents of mannitol or NaCl. The root and shoot lengths were measured, and no obvious differences were observed between the transgenic and WT plants on 1/2 MS medium. However, on medium with 100 mM NaCl, 200 mM mannitol, and 250 mM mannitol, *SiLTP* transgenic lines had significantly higher germination ratios than those of the WT (**Figures 4B,C**), and the *SiLTP* transgenic plants had longer roots and shoots on the media supplemented with either 100 mM NaCl or 200 mM mannitol compared than the WT seedlings (**Figures 4D,E**). To further determine the effect of abiotic stress on *SiLTP* transgenic tobacco, 4-week-old plants growing in soil were not irrigated with water, simulating drought stress (**Figure 5A**) or were irrigated with water supplemented with 250 mM NaCl (**Figure 6A**). After 3 weeks of the no irrigation drought treatment or 4 weeks of the salt treatment, the survival rates of these three transgenic lines were both significantly higher than those of WT plants (**Figures 5B and 6B**). Accordingly, transgenic lines also had significantly higher proline and soluble sugar contents than those of the WT under drought and salt treatments (**Figures 5C,D and 6C,D**). These results indicated that *SiLTP* improves the salt and drought tolerance of transgenic tobacco.

### *SiLTP* Participates in Salt and Drought Tolerance of Foxtail Millet

**FIGURE 7:**
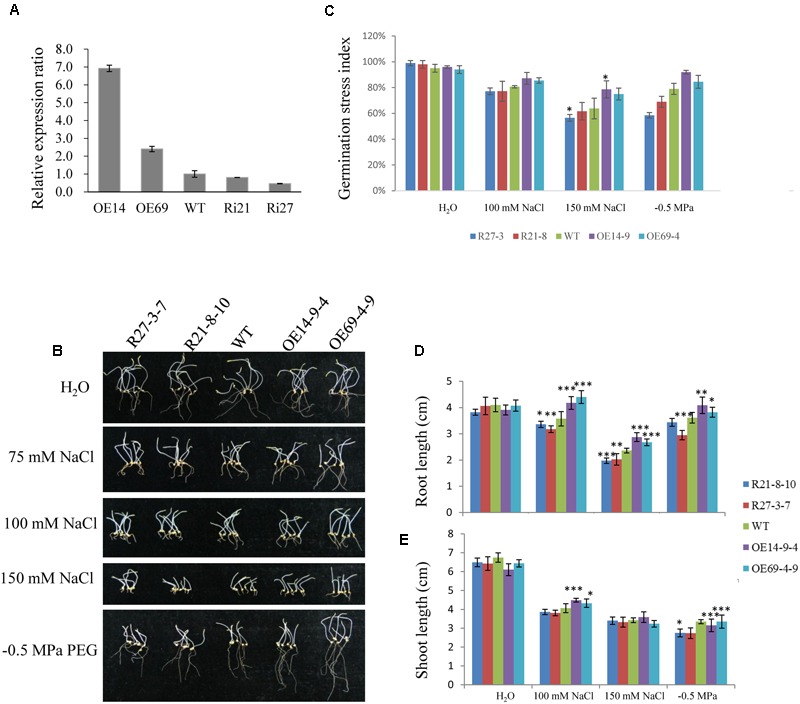
**Effects of NaCl and PEG stresses on foxtail millet germination rates.** Relative expression levels of SiLTP in T1 transgenic foxtail millet and WT plants as determined by qPCR **(A)**. Seeds of WT and transgenic lines were germinated and grown for 8 days in water independently supplemented with 0 (control), 100 mM, 150 mM NaCl, or -0.5 MPa PEG **(B)**. The corresponding seed germination rates **(C)**, root lengths **(D)**, and shoot lengths **(E)** were compared. ^∗^, ^∗∗^, and ^∗∗∗^ indicate statistically significant differences at *P* < 0.05, *P* < 0.01, and *P* < 0.001 (Student’s *t*-test), respectively.

For further analyzing *SiLTP* function in foxtail millet, OE and RNAi transgenic plants were generated. According to the result of qPCR, two OE lines (OE14 and OE69) and two RNAi lines (Ri21 and Ri27) were selected for further analysis (**Figure 7A**). The T2 transgenic seeds were used to analyze germination under normal conditions, and after salinity and drought treatments (**Figure 7B**). No significant germination stress indices changed among the OE, RNAi and WT lines (**Figure 7C**). However, after the independent 100 mM NaCl and -0.5 MPa PEG treatments, the roots and shoots of the OE lines were significantly longer than those of the WT and RNAi lines. Additionally, after the 150 mM NaCl treatment, the OE lines’ roots, but not the shoots, were significantly longer than in WT and RNAi lines (**Figures 7D,E**), the result was likely with that of *SiLTP* transgenic tobacco seeds on 250 mM mannitol treatment (**Figures 4D,E**).

**FIGURE 8:**
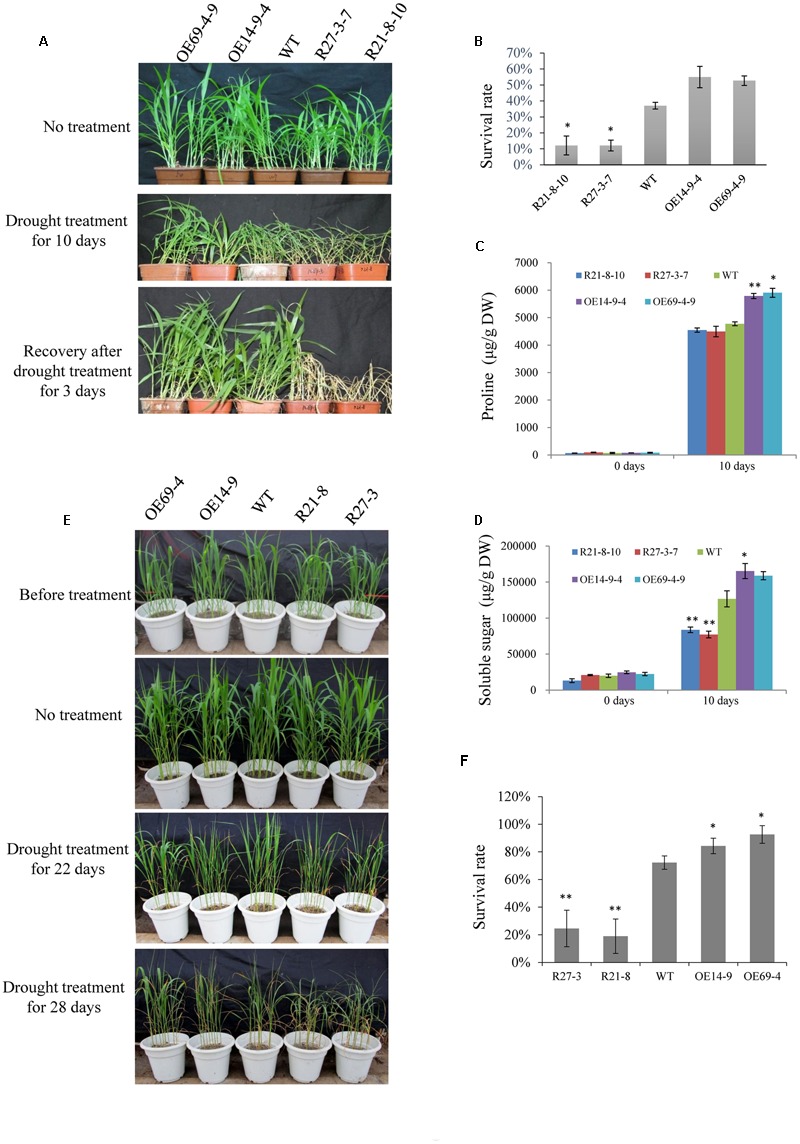
**Drought tolerance of *SiLTP-OE* and RNAi foxtail millet.** The phenotype of the 2-week-old transgenic foxtail millet seedlings under drought treatment **(A)**. The survival rates of transgenic foxtail millet and WT plants **(B)**. Proline contents **(C)** and soluble sugar contents **(D)** in WT and transgenic plants after drought stress. The drought tolerance phenotypes of *SiLTP-OE* and RNAi foxtail millet at the reproductive stage **(E)** and the survival rates of transgenic foxtail millet **(F)**. ^∗^ and ^∗∗^ indicate statistically significant differences at *P* < 0.05 and *P* < 0.01 (Student’s *t*-test), respectively.

**FIGURE 9:**
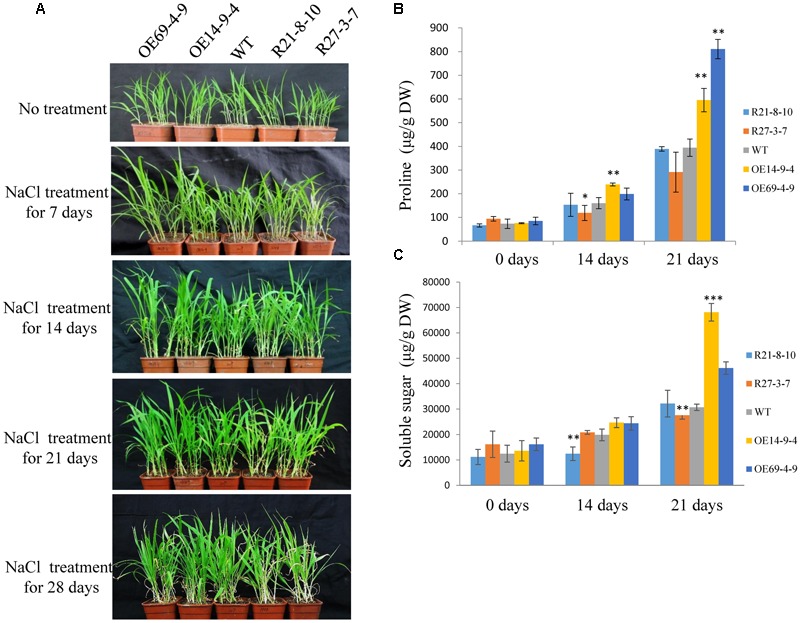
**Salt tolerance of *SiLTP-OE* and RNAi foxtail millet seedlings.** The phenotypes of transgenic foxtail millet under a 100 mM NaCl treatment **(A)**. Proline contents **(B)** and soluble sugar contents **(C)** were analyzed in WT and transgenic plants after watering for 14 and 21 days with NaCl, respectively. ^∗^, ^∗∗^, and ^∗∗∗^ indicate statistically significant differences at *P* < 0.05, *P* < 0.01, and *P* < 0.001 (Student’s *t*-test), respectively.

Furthermore, 2-week-old seedlings of WT and transgenic plants were not irrigated for 10 days, and were then re-irrigated and grown under normal conditions for 3 days. The survival rates of the OE lines were higher than that of the WT, and the RNAi lines had much lower survival rates compared with the WT (**Figures 8A,B**). The proline and soluble sugar contents of the OE lines (especially the OE14 line) were higher than those of WT and RNAi lines after 10 days of drought treatment, and the RNAi lines had significantly lower soluble sugar contents than those of the WT and OE lines (**Figures 8C,D**). Moreover, the drought treatment was implemented during the reproductive stage of foxtail millet grown in a greenhouse. After 22 days of drought treatment, RNAi lines had visually retarded growth rates than WT and OE lines (**Figure 8E**). After 28 days of the drought treatment, the survival rate of RNAi lines significantly decreased compared with those of OE lines and the WT, and the OE line survival rates were much higher than those of WT and RNAi lines (**Figures 8E,F**). When 2-week-old seedlings of WT and transgenic plants were irrigated with water supplemented with 100 mM NaCl, none of the seedlings showed any significant differences after 14 days of treatment (**Figure 9A**). *SiLTP*-OE seedlings showed more tolerance compared with those of WT and RNAi lines after 21 days and 28 days of treatments (**Figure 9A**). After 21 days of treatment, the proline and soluble sugar contents of *SiLTP*-OE line 14 were significantly greater than those of the WT and RNAi lines (**Figures 9B,C**). The *SiLTP*-OE 69 line had a significantly greater proline content compared with the WT and RNAi lines, and its soluble sugar content was also greater, but not significantly (**Figures 9B,C**).

Thus, the OE of *SiLTP* in foxtail millet enhanced salt and drought stress-related tolerance, while the down regulation of *SiLTP* led to an increased sensitivity to salt and drought stresses.

### SiARDP Regulates *SiLTP* Expression by Directly Binding the DRE Element

**FIGURE 10:**
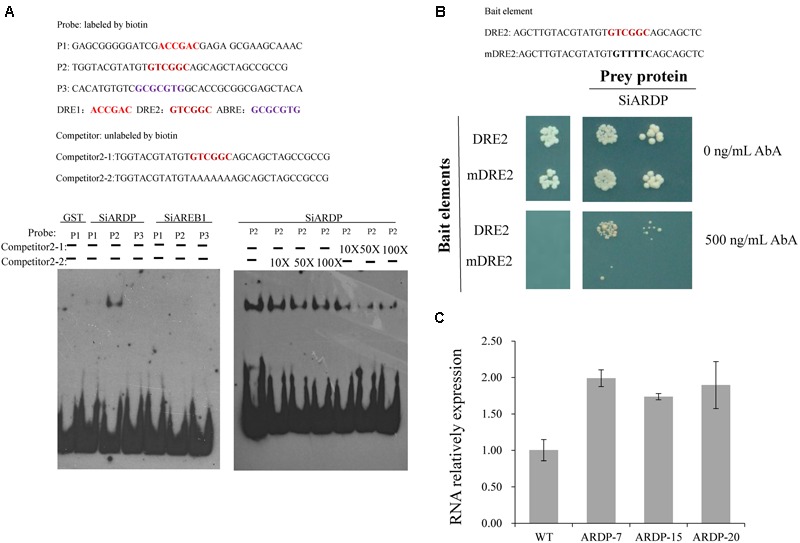
**SiARDP regulates SiLTP expression by directly binding to the DRE element.** EMSA for SiARDP and SiAREB1 binding to the promoter of SiLTP **(A)**; yeast one-hybrid assay for SiARDP binding to the DRE element of the SiLTP promoter **(B)** and the SiLTP expression levels in SiARDP transgenic foxtail millet **(C)**. Total RNA was extracted from 30 plants at the indicated times after the treatments. Data represent means and standard deviations for three biological replicates.

PlantCARE was used to analyze the promoter region of *SiLTP*, three *cis*-elements including two DRE and one AREB, were discovered (Supplementary Table S2) (http://bioinformatics.psb.ugent.be/webtools/plantcare/html/). EMSA was carried out and the result indicated that SiARDP bound to the *SiLTP* promoter through the DRE elements *in vitro*, but SiABRE1 could not bind to the AREB element (**Figure 10A**). Meanwhile, yeast one-hybrid results showed that SiARDP could bind to the *SiLTP* DRE elements *in vivo* (**Figure 10B**). The *SiLTP* transcription levels in *SiARDP*-OE lines were measured as well. The qPCR results showed that *SiLTP* was upregulated in *SiARDP*-OE lines (Li et al., 2014) by ∼1.5- to 2.0-fold compared with the WT (**Figure 10C**). Thus, *SiLTP* may be a downstream gene of SiARDP TF.

## Discussion

The nsLTPs are known for their ability to transfer different lipids *in vitro*, but their functions have not yet been elucidated *in vivo*. LTPs were subdivided into two families based on size, LTP I and LTP II. So far, functional studies of the LTP II family are still very limited. Most found that LTP II-type genes are associated with seed development (GarcćA-Garrido et al., 1998; Monnet et al., 2001; Castro et al., 2003). *LTP2* is an aleurone-specific expressed gene, and the *LTP2* promoter drove GUS specific expression in the aleurone layer of rice immature seeds (Kalla et al., 1994). A 7-kDa nsLTP2 isolated from rice (*O. sativa*) seeds (Liu et al., 2002) has an unclear function. Only two type II LTP genes that respond to drought stress have been cloned, and the expression was detected in leaves, stems, and crowns (Jang et al., 2002). Here, we reported the identification and characterization of *SiLTP*, a novel gene belonging to the LTP II family in foxtail millet, and this will help further the understanding of LTP II functions in abiotic stress responses.

Drought stress affects plant growth and development. In the early stages of drought stress, leaf and stem growth is suppressed, while root growth is increased (Sharp et al., 1988; Nonami and Boyer, 1990). The leaves and stems stop growing to preserve the limited carbohydrate content and maintain their own metabolism. Additionally, it enriches the insoluble substances in the cell to maintain osmotic balance (Osorio et al., 1998). Root elongation allows plants to absorb water from deeper soils to maintain their physiological activities (Munns et al., 2000). In this study, when seeds of *SiLTP* transgenic and WT tobacco were germinated on 1/2 MS medium containing 200 mM mannitol, the *SiLTP* transgenic plants had significantly longer roots and shoots compared than the WT (**Figure 4D**). As the mannitol content increased to 250 mM, both WT and transgenic plants were more seriously restrained with little changes in shoot length and significant difference of root length (**Figures 4D,E**). When 5-day-old tobacco seedlings were placed on the 1/2 MS medium that contained 200, 250, or 300 mM mannitol, the transgenic seedlings had longer root length than the WT, even though the roots and shoots of WT and transgenic seedlings were restrained (Supplementary Figure S1). In foxtail millet, the same trends in root and shoot elongation were observed under -0.5 MPa PEG treatments (**Figures 7D,E**). All these results implied that *SiLTP* may function in drought stress resistance through enhancing root elongation. Osmotic adjustment is an important way in which plants resist environmental stresses. Under salt and drought stresses, a large number of organic osmolytes, such as sugars, alcohols, amino acids, and betaine, accumulate in plant cells. These substances maintain inflation pressure and the cell’s structural stability (Yancey et al., 1982). Increases in the proline and sugar contents of cells can enhance the abiotic stress resistance of plants (Gilmour et al., 2000; Streeter et al., 2001; Roosens et al., 2002; Ashraf and Foolad, 2007). We found the similar results in *SiLTP* transgenic plants. The proline and soluble sugar contents in *SiLTP* transgenic tobacco plants were significantly greater than those of WT when treated with salt and drought stress (**Figures 5** and **6**). The proline contents of *SiLTP*-OE lines were also significant greater than that of WT and RNAi lines after 10 days drought treatment, while the soluble sugar contents of RNAi lines were significant lower than that of WT and OE lines (**Figures 8C,D**).

The plant hormone ABA has vital roles in the plant signaling pathways of abiotic stress responses (Danquah et al., 2013). *SiARDP*, a DREB-type TF, is upregulated by the AREB-type TFs, SiAREB1 and SiAREB2. SiAREB1, SiAREB2, and SiARDP are involved in the ABA-dependent signal transduction pathways, and can improve the salt and drought stress resistance of transgenic foxtail millet (Li et al., 2014). In this study, the *SiLTP* mRNA level was induced by the ABA treatment (**Figure 2C**), and the *SiLTP* promoter sequence contained two DRE elements. *SiARDP* and *SiLTP* have similar expression patterns under drought and ABA treatments. *SiLTP* expression was upregulated by ∼1.5- to 2.0-fold in the *SiARDP*-*OE* foxtail millet. In addition, an EMSA and a yeast one-hybrid assay showed interactions between SiARDP and the *SiLTP* promoter *in vitro* and *in vivo* (**Figures 10A,B**). All these implied that *SiLTP* may be a direct target of SiARDP during the positive regulation of plant responses to salt and drought stresses. MYB and MYC TFs are also important in responses to drought and salt stress (Agarwal and Jha, 2010). In *Arabidopsis, LTP3* expressed in various tissues and its expression is induced by cold, drought, and ABA. The LTP3 is localized in the cytoplasm. MYB96 directly binds to the *LTP3* promoter, regulating *LTP3* expression, and participating in the plant’s tolerance to freezing and drought stresses (Guo et al., 2013). In this study, *SiLTP* was induced by NaCl, drought, and ABA. *SiLTP* was expressed in all of the tested tissues, and SiLTP localized to the cytoplasm. The expression pattern and localization of *SiLTP* are similar to *LTP3*, and their regulation patterns may also be similar. In the *SiLTP* promoter region, we found five MYB and two MYC recognition sites (Supplementary Table S2). Therefore, it could be speculated that the MYB and MYC TFs take part in the regulation of *SiLTP* transcript levels except DREB type TFs. Further research is needed to investigate the regulation of *SiLTP*.

In transgenic tobacco and foxtail millet, *SiLTP* was proven to take part in the response to salt and drought stress, however, how the protein work in cells is unknown. When response to abiotic stress, cells would reduce lipid fluidity or decrease the membrane solute permeability to keep its integrity (Liu et al., 2015). Cryoprotectin, a LTP from cabbage, was proved to posse the cryoprotective activity without lipid transfer activity in cold treatment (Hincha et al., 2001). EARLI1 from *Arabidopsis*, can reduce electrolyte leakage during freezing damage, and was speculated to participate in the cell membrane or cell wall modification (Bubier and Schläppi, 2004). The Ca^2+^ sensor, calmodulin (CaM) is a mediator of intracellular Ca^2+^ signal transduction pathways (Golldack et al., 2014). Some evidence showed that LTPs can bind to CaM by their CaM-binding site (Wang et al., 2005, 2008; Gao et al., 2009), imply LTPs may through Ca^2+^ signal transduction pathways to participate in abiotic stress. Besides abiotic stress tolerance, some LTPs were found to possess biotic resistance or effects on plant growth (Sabine and Jutta, 2015; Safi et al., 2015). In our study, *SiLTP* RNAi lines showed slight morphological retardation in growth at the earlier stage under drought and salt treatments, we inferred that SiLTP may also participate in plant growth. Further research is needed to study the biological function of SiLTP.

In summary, *SiLTP* induced by NaCl, PEG, and ABA treatments and plays important roles in salt and drought stresses resistance in foxtail millet. *SiLTP* may be involved in ABA-dependent signaling pathway as a candidate of SiARDP downstream genes. This study provides insights into SiLTP and its regulator in plants response to abiotic stress, and a valid approach for improving abiotic stress resistance of crop.

## Author Contributions

YP and JY designed the experiments; YP, JL, and LJ did the experiments; YP completed the data analysis and wrote the original manuscript; CL constructed vectors and *SiARDP* transgenic plants; DZ planted the foxtail millet; JY modified the manuscript and finalize the manuscript. All authors consent to the final manuscript.

## Conflict of Interest Statement

The authors declare that the research was conducted in the absence of any commercial or financial relationships that could be construed as a potential conflict of interest.

## References

[B1] Abuqamar S. Luo H. Laluk K. Mickelbart M. V. Mengiste T. (2009). Crosstalk between biotic and abiotic stress responses in tomato is mediated by the aim1 transcription factor. *Plant J.* 58 347–360. 10.1111/j.1365-313X.2008.03783.x 19143995

[B2] Agarwal P. K. Jha B. (2010). Transcription factors in plants and ABA dependent and independent abiotic stress signaling. *Biol. Plant.* 54 201–212. 10.1007/s10535-010-0038-7

[B3] Arondel V. Kader J. C. (1990). Lipid transfer in plants. *Experientia* 46 579–585. 10.1007/BF01939696 2193821

[B4] Arnold K. Bordoli L. Kopp J. Schwede T. (2006). The SWISS-MODEL workspace: a web-based environment for protein structure homology modelling. *Bioinformatics* 22 195–201. 10.1093/bioinformatics/bti770 16301204

[B5] Ashraf M. Foolad M. R. (2007). Roles of glycine betaine and proline in improving plant abiotic stress resistance. *Environ. Exp. Bot.* 59 206–216. 10.1016/j.envexpbot.2005.12.006

[B6] Atkinson N. J. Lilley C. J. Urwin P. E. (2013). Identification of genes involved in the response of *Arabidopsis* to simultaneous biotic and abiotic stresses. *Plant Physiol.* 162 2028–2041. 10.1104/pp.113.222372 23800991 PMC3729780

[B7] Bates L. S. Waldren R. P. Teare I. D. (1973). Rapid determination of free proline for water-stress studies. *Plant Soil* 39 205–207. 10.1016/j.dental.2010.07.006

[B8] Bendtsen J. D. Nielsen H. von Heijne G. Brunak S. (2004). Improved prediction of signal peptides: SignalP 3.0. *J. Mol. Biol.* 340 783–795. 10.1016/j.jmb.2004.05.028 15223320

[B9] Bennetzen J. L. Schmutz J. Wang H. Percifield R. Hawkins J. Pontaroli A. C. (2012). Reference genome sequence of the model plant *Setaria*. *Nat. Biotechnol.* 30 555–561. 10.1038/nbt.2196 22580951

[B10] Bouslama M. Schapaugh W. T. (1984). Stress tolerance in soybeans. I. evaluation of three screening techniques for heat and drought tolerance. *Crop Sci.* 24 933–937.

[B11] Bubier J. Schläppi M. (2004). Cold induction of EARLI1 a putative *Arabidopsis* lipid transfer protein, is light and calcium dependent. *Plant Cell Environ.* 27 929–936. 10.1111/j.1365-3040.2004.01198.x

[B12] Bustin S. A. Benes V. Garson J. A. Hellemans J. Huggett J. Kubista M. (2009). The MIQE guidelines: minimum information for publication of quantitative real-time PCR experiments. *Clin. Chem.* 55 611–622. 10.1373/clinchem.2008.112797 19246619

[B13] Castagnaro A. García-Olmedo F. (1994). A fatty-acid-binding protein from wheat kernels. *FEBS Lett.* 349 117–119. 10.1016/0014-5793(94)00660-1 8045286

[B14] Castro M. S. Gerhardt I. R. Orrù S. Pucci P. Bloch C. (2003). Purification and characterization of a small (7.3 kDa) putative lipid transfer protein from maize seeds. *J. Chromatogr. B Analyt. Technol. Biomed. Life Sci.* 794 109–114. 10.1016/S1570-0232(03)00423-9 12888203

[B15] Chae K. Kieslich C. A. Morikis D. Kim S. C. Lord E. M. (2009). A gain-of-function mutation of *Arabidopsis* lipid transfer protein 5 disturbs pollen tube tip growth and fertilization. *Plant Cell* 21 3902–3914. 10.1105/tpc.109.070854 20044438 PMC2814499

[B16] Clough S. J. Bent A. F. (1998). Floral dip: a simplified method for *Agrobacterium*-mediated transformation of *Arabidopsis thaliana*. *Plant J.* 16 735–743. 10.1046/j.1365-313x.1998.00343.x 10069079

[B17] Danquah A. Zelicourt A. D. Colcombet J. Hirt H. (2013). The role of ABA and MAPK signaling pathways in plant abiotic stress responses. *Biotechnol. Adv.* 32 40–52. 10.1016/j.biotechadv.2013.09.006 24091291

[B18] Debono A. Yeats T. H. Rose J. K. Bird D. Jetter R. Kunst L. (2009). *Arabidopsis* LTPG is a glycosylphosphatidylinositol-anchored lipid transfer protein required for export of lipids to the plant surface. *Plant Cell* 21 1230–1238. 10.1105/tpc.108.064451 19366900 PMC2685631

[B19] Doust A. N. Kellogg E. A. Devos K. M. Bennetzen J. L. (2009). Foxtail millet: a sequence-driven grass model system. *Plant Physiol.* 149 137–141. 10.1104/pp.108.129627 19126705 PMC2613750

[B20] Edstam M. M. Laurila M. Hoglund A. Raman A. Dahlstrom K. M. Salminen T. A. (2014). Characterization of the GPI-anchored lipid transfer proteins in the moss *Physcomitrella patens*. *Plant Physiol. Biochem.* 75 55–69. 10.1016/j.plaphy.2013.12.001 24374350

[B21] Edstam M. M. Viitanen L. Salminen T. A. Edqvist J. (2011). Evolutionary history of the non-specific lipid transfer proteins. *Mol. Plant* 4 947–964. 10.1093/mp/ssr019 21486996

[B22] Gao G. Jin L. P. Xie K. Y. Qu D. Y. (2009). The potato StLTPa7 gene displays a complex Ca-associated pattern of expression during the early stage of potato-*Ralstonia solanacearum* interaction. *Mol. Plant Pathol.* 10 15–27. 10.1111/j.1364-3703.2008.00508.x 19161349 PMC6640406

[B23] GarcćA-Garrido J. M. Menossi M. Puigdoménech P. MartıìNez-Izquierdo J. A. Delseny M. (1998). Corrigendum to: characterization of a gene encoding an abscisic acid-inducible type-2 lipid transfer protein from rice. *FEBS Lett.* 428 193–199. 10.1016/S0014-5793(98)00529-8 9654133

[B24] Gilmour S. J. Sebolt A. M. Salazar M. P. Everard J. D. Thomashow M. F. (2000). Overexpression of the *Arabidopsis* CBF3 transcriptional activator mimics multiple biochemical changes associated with cold acclimation. *Plant Physiol.* 124 1854–1865. 10.1104/pp.124.4.1854 11115899 PMC59880

[B25] Golldack D. Li C. Mohan H. Probst N. (2014). Tolerance to drought and salt stress in plants: unraveling the signaling networks. *Front. Plant Sci.* 5:151 10.3389/fpls.2014.00151 PMC4001066 24795738

[B26] Guo C. Ge X. Ma H. (2013). The rice OsDIL gene plays a role in drought tolerance at vegetative and reproductive stages. *Plant Mol. Biol.* 82 239–253. 10.1007/s11103-013-0057-9 23686450

[B27] Han G. W. Lee J. Y. Song H. K. Chang C. Min K. Moon J. (2001). Structural basis of non-specific lipid binding in maize lipid-transfer protein complexes revealed by high-resolution X-ray crystallography. *J. Mol. Biol.* 308 263–278. 10.1006/jmbi.2001.4559 11327766

[B28] Hincha D. K. (2002). Cryoprotectin: a plant lipid-transfer protein homologue that stabilizes membranes during freezing. *Philos. Trans. R. Soc. B Biol. Sci.* 357 909–916. 10.1098/rstb.2002.1079 PMC1693006 12171654

[B29] Hincha D. K. Neukamm B. Sror H. A. Sieg F. Weckwarth W. Schmitt J. M. (2001). Cabbage cryoprotectin is a member of the nonspecific plant lipid transfer protein gene family. *Plant Physiol.* 125 835–846. 11161041 10.1104/pp.125.2.835 PMC64885

[B30] Hughes M. A. Dunn M. A. Pearce R. S. White A. J. Zhang L. (1992). An abscisic-acid-responsive, low temperature barley gene has homology with a maize phospholipid transfer protein. *Plant Cell Environ.* 15 861–865. 10.1111/j.1365-3040.1992.tb02155.x

[B31] Jang C. Kim D. Bu S. Kim J. Lee S. Kim J. (2002). Isolation and characterization of lipid transfer protein (LTP) genes from a wheat-rye translocation line. *Plant Cell Rep.* 20 961–966. 10.1007/s00299-001-0424-x

[B32] Jefferson R. A. Kavanagh T. A. Bevan M. W. (1987). GUS fusions: b-Glucuronidase as a sensitive and versatile gene fusion marker in higher plants. *EMBO J.* 6 3901–3907. 3327686 10.1002/j.1460-2075.1987.tb02730.x PMC553867

[B33] Jia G. Huang X. Zhi H. Zhao Y. Zhao Q. Li W. (2013). A haplotype map of genomic variations and genome-wide association studies of agronomic traits in foxtail millet (*Setaria italica*). *Nat. Genet.* 45 957–961. 10.1038/ng.2673 23793027

[B34] Kader J. C. (1975). Proteins and the intracellular exchange of lipids. I. Stimulation of phospholipid exchange between mitochondria and microsomal fractions by proteins isolated from potato tuber. *Biochim. Biophys. Acta* 380 31–44. 804327

[B35] Kader J. C. (1996). Lipid-transfer proteins in plants. *Annu. Rev. Plant Biol.* 47 627–654. 10.1146/annurev.arplant.47.1.627 15012303

[B36] Kalla R. Shimamoto K. Potter R. Nielsen P. S. Linnestad C. Olsen O. A. (1994). The promoter of the barley aleurone-specific gene encoding a putative 7 kDa lipid transfer protein confers aleurone cell-specific expression in transgenic rice. *Plant J.* 6 849–860. 10.1046/j.1365-313X.1994.6060849.x 7849757

[B37] Kim H. Lee S. B. Kim H. J. Min M. K. Hwang I. Suh M. C. (2012). Characterization of glycosylphosphatidylinositol-anchored lipid transfer protein 2 (LTPG2) and overlapping function between LTPG/LTPG1 and LTPG2 in cuticular wax export or accumulation in *Arabidopsis thaliana*. *Plant Cell Physiol.* 53 1391–1403. 10.1093/pcp/pcs083 22891199

[B38] Kumar K. Muthamilarasan M. Prasad M. (2013). Reference genes for quantitative real-time PCR analysis in the model plant foxtail millet (*Setaria, italica*, L.) subjected to abiotic stress conditions. *Plant Cell Tissue Organ Cult.* 115 13–22. 10.1007/s11240-013-0335-x

[B39] Li C. Yue J. Wu X. Xu C. Yu J. (2014). An ABA-responsive DRE-binding protein gene from *Setaria italica*, SiARDP, the target gene of SiAREB, plays a critical role under drought stress. *J. Exp. Bot.* 65 5415–5427. 10.1093/jxb/eru302 25071221 PMC4157718

[B40] Liu F. Zhang X. Lu C. Zeng X. Li Y. Fu D. (2015). Non-specific lipid transfer proteins in plants: presenting new advances and an integrated functional analysis. *J. Exp. Bot.* 66 5663 10.1093/jxb/erv313 26139823

[B41] Liu Y. J. Samuel D. Lin C. H. Lyu P. C. (2002). Purification and characterization of a novel 7-kDa non-specific lipid transfer protein-2 from rice (*Oryza sativa*). *Biochem. Biophys. Res. Commun.* 294 535–540. 10.1016/S0006-291X(02)00509-0 12056799

[B42] Longeman J. Schell J. Willmitzer L. (1987). Improved method for the isolation of RNA from plant tissues. *Anal. Biochem.* 163 16–20. 10.1016/0003-2697(87)90086-8 2441623

[B43] Mahajan S. Tuteja N. (2005). Cold, salinity and drought stresses: an overview. *Arch. Biochem. Biophys.* 444 139–158. 10.1016/j.abb.2005.10.018 16309626

[B44] Maldonado A. M. Doerner P. Dixon R. A. Lamb C. J. Cameron R. K. (2002). A putative lipid transfer protein involved in systemic resistance signalling in *Arabidopsis*. *Nature* 419 399–403. 10.1038/nature00962 12353036

[B45] Monnet F. P. Dieryck W. Boutrot F. Joudrier P. Gautier M. F. (2001). Purification, characterisation and cDNA cloning of a type 2 (7 kDa) lipid transfer protein from *Triticumdurum*. *Plant Sci.* 161 747–755. 10.1016/S0168-9452(01)00459-9

[B46] Munns R. Passioura J. B. Guo J. Chazen O. Cramer G. R. (2000). Water relations and leaf expansion: importance of time scale. *J. Exp. Bot.* 51 1495–1504. 10.1093/jexbot/51.350.1495 11006301

[B47] Muthamilarasan M. Prasad M. (2015). Advances in Setaria genomics for genetic improvement of cereals and bioenergy grasses. *Theor. Appl. Genet.* 128 1–14. 10.1007/s00122-014-2399-3 25239219

[B48] Nonami H. Boyer J. S. (1990). Primary events regulating stem growth at low water potentials. *Plant Physiol.* 93 1601–1609. 10.1104/pp.93.4.1601 16667663 PMC1062718

[B49] Osorio J. Osorio M. L. Chaves M. M. Pereira J. S. (1998). Water deficits are more important in delaying growth than in changing patterns of carbon allocation in *Eucalyptus globulus*. *Tree Physiol.* 18 363–373. 10.1093/treephys/18.6.363 12651361

[B50] Pan Y. Ma X. Liang H. Zhao Q. Zhu D. Yu J. (2015). Spatial and temporal activity of the foxtail millet (*Setaria italica*) seed-specific promoter pF128. *Planta* 241 57–67. 10.1007/s00425-014-2164-5 25204632

[B51] Panaud O. (2006). *Foxtail Millet. In Cereals and Millets.* Berlin: Springer 325–332.

[B52] Pitzschke A. Datta S. Persak H. (2014). Salt stress in *Arabidopsis*: lipid transfer protein AZI1 and its control by mitogen-activated protein kinase MPK3. *Mol. Plant* 7 722–738. 10.1093/mp/sst157 24214892 PMC3973493

[B53] Prasad M. Lata C. Yadav A. (2011). “Role of plant transcription factors in abiotic stress tolerance,” in *Abiotic Stress Response in Plants – Physiological, Biochemical and Genetic Perspectives* eds Shanker A. K. Venkateswarlu B. (Rijeka: InTech Press) 269–296.

[B54] Pyee J. Yu H. Kolattukudy P. E. (1994). Identification of a lipid transfer protein as the major protein in the surface wax of broccoli (*Brassica oleracea*) leaves. *Arch. Biochem. Biophys.* 311 460–468. 10.1006/abbi.1994.1263 8203911

[B55] Qin F. Zhao Q. Ao G. Yu J. (2008). Co-suppression of Si401 a maize pollen specific Zm401 homologous gene, results in aberrant anther development in foxtail millet. *Euphytica* 163 103–111. 10.1007/s10681-007-9610-4

[B56] Roosens N. H. Bitar F. Loenders K. Angenon G. Jacobs M. (2002). Overexpression of ornithine-δ-aminotransferase increases proline biosynthesis and confers osmotolerance in transgenic plants. *Mol. Breed.* 9 73–80. 10.1023/A:1026791932238

[B57] Sabine J. Jutta L. M. (2015). Response of *Arabidopsis thaliana* roots with altered lipid transfer protein (ltp) gene expression to the clubroot disease and salt stress. *Plants* 5:2 10.3390/plants5010002 PMC4844412 27135222

[B58] Safi H. Saibi W. Alaoui M. M. Hmyene A. Masmoudi K. Hanin M. (2015). A wheat lipid transfer protein (TDLTP4) promotes tolerance to abiotic and biotic stress in *Arabidopsis thaliana*. *Plant Physiol. Biochem.* 89C 64–75. 10.1016/j.plaphy.2015.02.008 25703105

[B59] Sham A. Alazzawi A. Alameri S. Almahmoud B. Awwad F. Alrawashdeh A. (2014). Transcriptome analysis reveals genes commonly induced by botrytis cinerea infection, cold, drought and oxidative stresses in *Arabidopsis*. *PLoS ONE* 9:e113718 10.1371/journal.pone.0113718 PMC4244146 25422934

[B60] Sham A. Moustafa K. Al-Ameri S. Al-Azzawi A. Iratni R. Abuqamar S. (2015). Identification of *Arabidopsis* candidate genes in response to biotic and abiotic stresses using comparative microarrays. *PLoS ONE* 10:e0125666 10.1371/journal.pone.0125666 PMC4416716 25933420

[B61] Sharp R. E. Silk W. K. Hsiao T. C. (1988). Growth of the maize primary root at low water potentials: I. Spatial distribution of expansive growth. *Plant Physiol.* 87 50–57. 16666126 10.1104/pp.87.1.50 PMC1054698

[B62] Silverstein K. A. Moskal W. J. Wu H. C. Underwood B. A. Graham M. A. Town C. D. (2007). Small cysteine-rich peptides resembling antimicrobial peptides have been under-predicted in plants. *Plant J.* 51 262–280. 10.1111/j.1365-313X.2007.03136.x 17565583

[B63] Streeter J. G. Lohnes D. G. Fioritto R. J. (2001). Patterns of pinitol accumulation in soybean plants and relationships to drought tolerance. *Plant Cell Environ.* 24 429–438. 10.1046/j.1365-3040.2001.00690.x

[B64] Thoma S. Kaneko Y. Somerville C. (1993). A non-specific lipid transfer protein from *Arabidopsis* is a cell wall protein. *Plant J.* 3 427–436. 10.1046/j.1365-313X.1993.t01-25-00999.x 8220451

[B65] Trevino M. B. O’Connell M. A. (1998). Three drought-responsive members of the nonspecific lipid-transfer protein gene family in Lycopersicon pennellii show different developmental patterns of expression. *Plant Physiol.* 116 1461–1468. 10.1104/pp.116.4.1461 9536064 PMC35054

[B66] Wang C. Xie W. Chi F. Hu W. Mao G. Sun D. (2008). BcLTP, a novel lipid transfer protein in *Brassica* chinensis, may secrete and combine extracellular CaM. *Plant Cell Rep.* 27 159–169. 10.1007/s00299-007-0434-4 17891402

[B67] Wang M. Li P. Li C. Pan Y. Jiang X. Zhu D. (2014). SiLEA14 a novel atypical LEA protein, confers abiotic stress resistance in foxtail millet. *BMC Plant Biol.* 14:290 10.1186/s12870-014-0290-7 PMC4243736 25404037

[B68] Wang M. Pan Y. Li C. Liu C. Zhao Q. Ao G. (2011). Culturing of immature inflorescences and *Agrobacterium*-mediated transformation of foxtail millet (*Setaria italica*). *Afr. J. Biotechnol.* 10 16466–16479.

[B69] Wang Z. Xie W. Chi F. Li C. (2005). Identification of non-specific lipid transfer protein-1 as a calmodulin-binding protein in *Arabidopsis*. *FEBS Lett.* 579 1683–1687. 10.1016/j.febslet.2005.02.024 15757661

[B70] Wu G. Robertson A. J. Liu X. Zheng P. Wilen R. W. Nesbitt N. T. (2004). A lipid transfer protein gene BG-14 is differentially regulated by abiotic stress, ABA, anisomycin, and sphingosine in bromegrass (*Bromus inermis*). *J. Plant Physiol.* 161 449–458. 15128032 10.1078/0176-1617-01259

[B71] Xu Z. Y. Zhang X. Schlappi M. Xu Z. Q. (2011). Cold-inducible expression of AZI1 and its function in improvement of freezing tolerance of *Arabidopsis thaliana* and *Saccharomyces cerevisiae*. *J. Plant Physiol.* 168 1576–1587. 10.1016/j.jplph.2011.01.023 21492954

[B72] Yamaguchi-Shinozaki K. Shinozaki K. (2005). Organization of cis-acting regulatory elements in osmotic- and cold-stress-responsive promoters. *Trends Plant Sci.* 10 88–94. 10.1016/j.tplants.2004.12.012 15708346

[B73] Yamaguchi-Shinozaki K. Shinozaki K. (2006). Transcriptional regulatory networks in cellular responses and tolerance to dehydration and cold stresses. *Annu. Rev. Plant Biol.* 57 781–803. 10.1146/annurev.arplant.57.032905.105444 16669782

[B74] Yancey P. H. Clark M. E. Hand S. C. Bowlus R. D. Somero G. N. (1982). Living with water stress: evolution of osmolyte systems. *Science* 217 1214–1222. 10.1126/science.7112124 7112124

[B75] Yemm E. W. Willis A. J. (1954). The estimation of carbohydrates in plant extracts by anthrone. *Biochem. J.* 57 508–514. 10.1042/bj0570508 13181867 PMC1269789

[B76] Yu K. Soares J. M. Mandal M. K. Wang C. Chanda B. Gifford A. N. (2013). A feedback regulatory loop between G3P and lipid transfer proteins DIR1 and AZI1 mediates azelaic-acid-induced systemic immunity. *Cell Rep.* 3 1266–1278. 10.1016/j.celrep.2013.03.030 23602565

[B77] Zhang D. Liang W. Yin C. Zong J. Gu F. Zhang D. (2010). OsC6 encoding a lipid transfer protein, is required for postmeiotic anther development in rice. *Plant Physiol.* 154 149–162. 10.1104/pp.110.158865 20610705 PMC2938136

[B78] Zhang G. Liu X. Quan Z. Cheng S. Xu X. Pan S. (2012). Genome sequence of foxtail millet (*Setaria italica*) provides insights into grass evolution and biofuel potential. *Nat. Biotechnol.* 30 549–554. 10.1038/nbt.2195 22580950

[B79] Zhang Y. Zhang X. Niu S. Han C. Yu J. Li D. (2010). Nuclear localization of beet black scorch virus capsid protein and its interaction with importin α. *Virus Res.* 155 307–315. 10.1016/j.virusres.2010.10.029 21056066

