# Supplementary material for: A Non-specific Setaria italica Lipid Transfer Protein Gene Plays a Critical Role under Abiotic Stress
Source: Front Plant Sci. 2016 Nov 24;7:1752. doi: 10.3389/fpls.2016.01752 (PMC5121218; doi:10.3389/fpls.2016.01752)
Supplement: Supplementary file 1 [file Presentation_1.PDF]

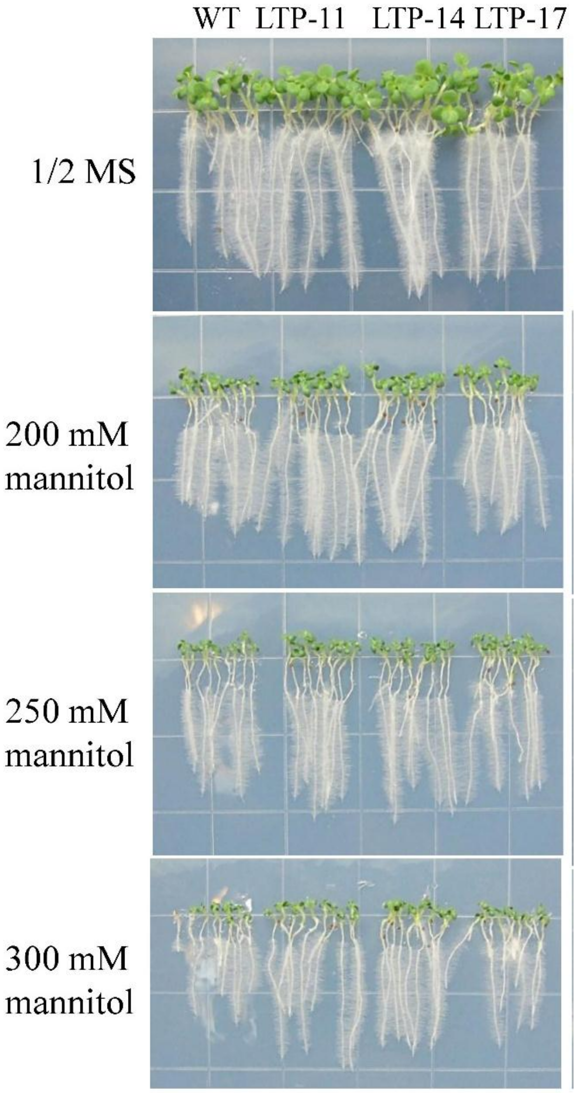

Supplemental Figure S1 Drought resistance analysis of T1 transgenic and WT tobacco seedlings. Five day old seedlings were put on the 1/2MS mediums which contained 0, 200, 250, and 300 mM mannitol, vertical placed in incubator, cultivate for 7 days. Each treatment were performed with three replicates.

Supplemental Table S1 Primers used in this article

Name of primer	Sequence(5' to3')	use
RT-ltp-1	AAGATGACGATGAAGCAGCAG	*SiLTP* gene
RT-ltp-2	GTTGGGGCTGTTGATGTAGC	For QRT-PCR and semi-RT-PCR
Actin1	TTGCTGACAGGATGAGCAAG	*Actin* gene (foxtail millet)
Actin2	CACATCTGCTGGAATGTGCT	For QRT-PCR and semi-RT-PCR
Actin3	GAACCAGAAGGATGCATATGTTG	*Actin* gene (tobacco)
Actin4	GGTAATCAGTAAGGTCACGTCCA	For semi-RT-PCR
LTP-*Xba* I	GCTCTAGAGCATGACGATGAAGC	*SiLTP* gene
LTP-*Kpn* I	GGTACCGCAGCGAGGGAC	For subcellular localization
pLTP-*Sal* I	CTGTCGACGCTGGGCTCCTTGCTGCTTTTC	*SiLTP* promoter amplification
pLTP-*Eco*R I	CCGGAATTCTCACTGTCACTGGTGGTG	
P1-R	GAGCGGGGGATCGACCGACGAGAGCGAAGCAAAC	Probe1: labeled by biotin
P1-F	GTTTGCTTCGCTCTCGTCGGTCGATCCCCCGCTC	For EMSA
P2-R	TGGTACGTATGTGTCGGCAGCAGCTAGCCGCCG	Probe2: labeled by biotin
P2-F	CGGCGGCTAGCTGCTGCCGACACATACGTACCA	For EMSA
P3-R	CACATGTGTCGCGCGTGGCACCGCGGCGAGCTACA	Probe3: labeled by biotin
P3-F	TGTAGCTCGCCGCGGTGCCACGCGCGACACATGTG	For EMSA
Competitor2-1R	TGGTACGTATGTGTCGGCAGCAGCTAGCCGCCG	Competitor2-1: unlabeled by biotin
Competitor2-1F	CGGCGGCTAGCTGCTGCCGACACATACGTACCA -	For EMSA
Competitor2-2R	TGGTACGTATGTAAAAAAAGCAGCTAGCCGCCG	Competitor2-2: unlabeled by biotin
Competitor2-2F	CGGCGGCTAGCTGCTTTTTTTACATACGTACCA	For EMSA
DRE2-F	AGCTTGTACGTATGTGTCGGCAGCAGCTC	Bait element
DRE2-R	TCGAGAGCTGCTGCCGACACATACGTACA	For yeast one-hybrid assay
m-DRE2-F	AGCTTGTACGTATGTGTTTTCAGCAGCTC	Bait element (mutant)
m-DRE2-R	TCGAGAGCTGCTGAAAACACATACGTACA	For yeast one-hybrid assay

Supplemental Table S2 Putative cis-acting regulatory elements associated with ABA and various stresses in *SiLTP* promoter region

Cis-element	Sequence	position	Function
ABRE	GCGCGTG	-175	ABA-mediated regulation of transcription
DRE	ACCGAC	-763	"DRE2" core found in maize (Z.M.) *rab17* gene promoter
	GTCGGC	-221	cis-acting element involved in dehydration, low-temp, salt stresses
MYB	TAACTG	-1280	MYB binding site involved in flavonoid biosynthetic genes regulation
	CAACGG	-1173	MYB binding site involved in drought-inducibility
	CGGTTA	-908	Myb binding site on the rd29B gene
	CGGTTG	-602<br>-539	MYB binding site involved in drought-inducibility
MYC	CAGCTC	-401	recognition site found in the promoters of the dehydration-responsive gene rd22 and many other genes in *Arabidopsis*
	CATGTG	-183	MYC recognition sequence (from -466 to -461) necessary for expression of *erd1* (early responsive to dehydration) in dehydrated *Arabidopsis*
